# Poor Mobilization-Associated Factors in Autologous Hematopoietic Stem Cell Harvest

**DOI:** 10.3390/cancers16101821

**Published:** 2024-05-10

**Authors:** Won Kee Ahn, Hyun-Jun Nam, Hae Won Lee, Seungmin Hahn, Jung Woo Han, Chuhl Joo Lyu, Sinyoung Kim, Soon Sung Kwon, Haerim Chung, Jin Seok Kim, June-Won Cheong, Kyung-A Lee

**Affiliations:** 1 Division of Pediatric Hematology and Oncology, Department of Pediatrics, Yonsei University College of Medicine, Yonsei University Health System, Seoul 03722, Republic of Korea; 2 Department of Laboratory Medicine, Yonsei University College of Medicine, Seoul 03722, Republic of Korea; 3 Division of Hematology, Department of Internal Medicine, Yonsei University College of Medicine, Seoul 03722, Republic of Korea

**Keywords:** peripheral blood stem cell harvest, hematologic malignancies, non-hematologic malignancies, plerixafor, poor mobilization

## Abstract

**Simple Summary:**

This study aims to describe the factors associated with poor mobilization in patients undergoing autologous peripheral blood stem cell harvest. Poor mobilization was observed in 30.5% of the patients included in the investigation. While older age, myelotoxic agents, and low platelet counts were associated with poor mobilization in patients with hematologic diseases, a history of radiation therapy to the spine and pelvic region was associated with mobilization failure in patients with non-hematologic diseases. In patients who received plerixafor, 14.2% suffered from mobilization failure, with differences in hemoglobin, platelet counts, and mobilization regimens noted between the poor and good mobilization groups. Identifying potential risk factors for poor mobilization in specific groups of patients will aid in planning therapy and ensuring successful transplantation in those requiring hematopoietic stem cell transplants.

**Abstract:**

Peripheral blood stem cell transplantation (PBSCT) is an important therapeutic measure for both hematologic and non-hematologic diseases. For PBSCT to be successful, sufficient CD34^+^ cells need to be mobilized and harvested. Although risk factors associated with poor mobilization in patients with hematologic diseases have been reported, studies of patients with non-hematologic diseases and those receiving plerixafor are rare. To identify factors associated with poor mobilization, data from autologous PBSC harvest (PBSCH) in 491 patients were retrospectively collected and analyzed. A multivariate analysis revealed that in patients with a hematologic disease, an age older than 60 years (odds ratio [OR] 1.655, 95% confidence interval [CI] 1.049–2.611, *p* = 0.008), the use of myelotoxic agents (OR 4.384, 95% CI 2.681–7.168, *p* < 0.001), and a low platelet count (OR 2.106, 95% CI 1.205–3.682, *p* = 0.009) were associated with poor mobilization. In patients with non-hematologic diseases, a history of radiation on the pelvis/spine was the sole associated factor (OR 12.200, 95% CI 1.934–76.956, *p* = 0.008). Among the group of patients who received plerixafor, poor mobilization was observed in 19 patients (19/134, 14.2%) and a difference in the mobilization regimen was noted among the good mobilization group. These results show that the risk factors for poor mobilization in patients with non-hematologic diseases and those receiving plerixafor differ from those in patients with hematologic diseases; as such, non-hematologic patients require special consideration to enable successful PBSCH.

## 1. Introduction

Peripheral blood stem cell transplantation (PBSCT) is an important therapeutic measure for patients with various clinical conditions, including hematologic malignancies, solid cancers, and inborn errors of immunity. Before PBSCT, a peripheral blood stem cell harvest (PBSCH) is performed using apheresis techniques to collect the hematopoietic stem cells (HSCs) to be transplanted. Both autologous and allogeneic PBSCT can be used depending on the individual patient’s condition and treatment objectives. Studies of the outcomes of PBSCT have been conducted based on the number of infused CD34^+^ cells, and 2–5 × 10^6^ cells/kg of the recipient’s body weight has been suggested as the optimal collection amount for a successful transplantation outcome [1,2,3]. Because the collection of sufficient CD34^+^ cells is important for successful transplantation, CD34^+^ cell mobilization using G-CSF and chemotherapy prior to PBSCH is a pivotal step.

In autologous PBSCH, cells are collected from patients themselves. Therefore, autologous PBSCH has a higher incidence of collection failure than allogeneic PBSCH, which harvests cells from healthy donors. Previous studies reported that the incidence of poor mobilization ranged from 15–32% [4,5,6,7]. When sufficient CD34^+^ cells cannot be collected, the patient must either undergo re-harvest or be treated with other options. These cause inconvenience and sometimes worsen clinical outcomes [8,9,10,11]. Previous studies have reported various factors associated with mobilization failure, and some researchers have developed methods to predict CD34^+^ cell collection yield [12,13,14,15,16,17,18,19].

Despite the numerous studies conducted on PBSCH, most have focused on patients with hematologic malignancies. Although a large proportion of patients undergoing PBSCH have hematologic malignancies, a significant number of patients undergo PBSCH for other reasons [20,21]. Therefore, investigating the failure rates and factors associated with failure in those patient groups is beneficial. The aim of this study was to investigate factors associated with mobilization failure in patients undergoing PBSCH. The patients included those with hematologic and non-hematologic diseases. Cases in which mobilization failure occurred despite the use of plerixafor were also included.

## 2. Materials and Methods

### 2.1. Patients

Clinical data and laboratory test results of patients who underwent autologous PBSCH at a tertiary hospital in Seoul, South Korea, between January 2018 and October 2023 were collected and analyzed retrospectively. This study was reviewed and approved by the Institutional Review Board (IRB) of Yonsei University Health System (4-2023-0875). The need for informed consent was waived by the IRB because this study was retrospective.

### 2.2. Mobilization and Collection Procedures

All patients received chemotherapy as part of our institution’s disease-specific protocol. Following the completion of the last cycle, G-CSF treatment was initiated immediately before bone marrow recovery. In patients with a hematologic disease and those whose protocol excluded alkylating drugs, G-CSF alone was used for mobilization. For patients with hematologic malignancies involving alkylating agents and all patients with non-hematologic malignancies, chemotherapy plus G-CSF therapy were utilized for mobilization. Mobilization chemotherapy regimens were grouped and included cyclophosphamide (≥2000 mg/m^2^), etoposide (≥300 mg/m^2^), cyclophosphamide plus etoposide, and high-dose cytarabine groups. A detailed mobilization chemotherapy regimen is demonstrated in Supplementary Table S1.

Plerixafor was given in cases of expected harvest failure to adult patients with non-Hodgkin lymphoma (NHL) or multiple myeloma (MM) and to pediatric patients with NHL or solid tumors. The expected failure criteria were a peripheral blood CD34^+^ cell count below 10 cells/μL or a harvest product CD34^+^ cell count below 0.7 × 10^6^ cells/kg for NHL patients and a count below 15 cells/μL or below 1.0 × 10^6^ cells/kg for adults with MM and pediatric patients with solid tumors.

Stem cell collection procedures were conducted using Spectra Optia (Terumo BCT, Tokyo, Japan) with the continuous mononuclear cell collection program. The total processed volume was 2–4 times the total blood volume. For anticoagulation, acid citrate dextrose solution-A (ACD-A) mixed with heparin (3000 IU/500 mL ACD-A) was used in a 1:24 ratio to blood.

### 2.3. Variables and Definition

We collected the following clinical data: demographics, diagnoses (classified as hematologic and non-hematologic disease), number of chemotherapy cycles and drugs used, laboratory parameters, bone marrow biopsy findings, use of mobilization chemotherapy or G-CSF alone, and a recent history of pelvic or spinal radiotherapy.

For the analysis of factors associated with poor mobilization, continuous values were categorized into two groups. Cutoff values for categorization were determined based on the median value of each variable. The myelotoxic agents were doxorubicin, lenalidomide, and melphalan. Poor mobilization was defined as either the use of plerixafor or the failure to collect 2.0 × 10^6^ CD34^+^ cells/kg throughout the entire harvest duration. In the plerixafor use group, failure to collect 2.0 × 10^6^ CD34^+^ cells/kg was defined as poor mobilization.

### 2.4. Statistics

For continuous variables, data are presented as medians with interquartile ranges (IQRs). For categorical variables, frequencies with percentages are presented. If appropriate, unpaired *t*-tests and Mann–Whitney U tests were used to compare differences in continuous variables between the poor and good mobilization groups. For categorical variables, chi-square tests and Fisher’s exact tests were performed to compare differences between groups. A logistic regression was conducted to identify factors associated with poor mobilization. Factors that were significantly associated with poor mobilization in the univariate analyses were subsequently included in the multivariable analysis. All statistical analyses were conducted using SPSS version 26.0 (IBM Corp., Armonk, NY, USA) with *p*-values of <0.05 considered statistically significant.

## 3. Results

### 3.1. Patient Characteristics

We analyzed data from 491 patients who underwent autologous PBSCH. The characteristics of the patients are detailed in Table 1. Among them, 419 patients underwent autologous PBSCH due to hematologic diseases, and 72 patients underwent autologous PBSCH to treat non-hematologic diseases. Among the 150 poor mobilizers (30.5%), 144 patients had hematologic diseases and 6 had non-hematologic diseases. Detailed diagnostic information is summarized in Supplementary Tables S2 and S3. The median age of the poor mobilizers was 60 years; 144 of these patients (96.0%) were older than 20 years and 74 patients (49.3%) were older than 60 years. In the poor mobilization group, the median number of chemotherapy cycles was five (IQR 4–7), and 102 patients (68.0%) were treated with myelotoxic agents. Fourteen patients (9.3%) had a history of radiation therapy that included the spinal and/or pelvic region prior to the autologous PBSCH. Bone marrow involvement at the time of harvest was identified in 22 patients (14.7%). On the day mobilization was initiated, hemoglobin and white blood cell (WBC) counts did not differ significantly between the poor and good mobilization groups; but the platelet count was significantly lower in the poor mobilization group. Mobilization with G-CSF alone was used in 111 patients (74.0%), and chemotherapy plus G-CSF mobilization was used in 39 patients (26.0%). The median number of peripheral CD34^+^ cells on day 1 of harvest was 5/μL (IQR 2–8), and the number harvested on day 1 was 0.45 × 10^6^/kg (IQR 0.20–0.71). Plerixafor was used in 134 patients (89.3%).

### 3.2. Factors Associated with Poor Mobilization in Patients with Hematologic and Non-Hematologic Diseases

For all patients included in this study, factors associated with poor mobilization were investigated (Table 2). In the univariate analysis, an age older than 20 years, the presence of hematologic disease, a number of chemotherapy cycles >6, the use of myelotoxic agents, a platelet count <150,000/μL on mobilization day 1, and a diagnosis to mobilization period of >200 days were significantly associated with poor mobilization. Among those factors, an age older than 20 years (odds ratio [OR] 4.336, a 95% confidence interval [CI] 1.131–16.623, *p* = 0.032), the use of myelotoxic agents (OR 4.093, 95% CI 2.571–6.516, *p* < 0.001), and a platelet count <150,000/μL (OR 2.687, 95% CI 1.624–4.445, *p* < 0.001) on mobilization day 1 were associated with poor mobilization in the multivariate analysis.

Due to differences in the characteristics of patients with hematologic diseases and those with non-hematologic diseases, factors associated with poor mobilization were analyzed in each patient group. The detailed characteristics of each group are summarized in Supplementary Table S4. In the hematologic disease group, the factors associated with poor mobilization were an age older than 60 years (OR 1.655, 95% CI 1.049–2.611, *p* = 0.030), the use of myelotoxic agents (OR 4.384, 95% CI 2.681–7.168, *p* < 0.001), and platelet count <150,000/μL on mobilization day 1 (OR 2.106, 95% CI 1.205–3.682, *p* = 0.009, Table 3).

Intriguingly, factors associated with poor mobilization in patients with hematologic diseases did not show associations with poor mobilization in those with non-hematologic diseases. A history of radiation therapy on the spine and/or pelvic region was the sole factor associated with poor mobilization in patients with non-hematologic diseases (OR 12.200, 95% CI 1.934–76.956, *p* = 0.008, Table 4).

### 3.3. Mobilization Failure in the Plerixafor Treatment Group

Patients whose PBSCH was predicted to fail based on the results of harvest day 1 received plerixafor. Of the 134 patients treated with plerixafor, PBSCH failed in 19 patients (14.2%). Among the factors included in the analysis, the hemoglobin and platelet counts on mobilization day 1 and the mobilization regimen differed significantly between the poor and good mobilization groups (Table 5).

## 4. Discussion

Studies of PBSCH have reported several factors associated with poor mobilization [12,13,14,15,16,17,18,19]: older age, low hemoglobin level, low platelet count, low WBC count, mobilization regimen, diagnosis of NHL, bone marrow involvement, previous PBSCH failure, previous use of myelotoxic agents, number of chemotherapy cycles, time from diagnosis to harvest, bone marrow cellularity, and peripheral CD34^+^ cell count. Among these factors, the pre-harvest peripheral CD34^+^ cell count measured after mobilization is the strongest indicator of the overall CD34^+^ cell yield. Thus, many groups use pre-harvest peripheral CD34^+^ cell count to predict mobilization failure. Despite its high correlation, pre-harvest peripheral CD34^+^ cell count is a result of mobilization rather than a predictor of collection yield. To make a timely decision at the beginning of mobilization, indicators other than the pre-harvest peripheral CD34^+^ cell count may be more useful. For example, an earlier study suggested a scoring system that used patient characteristics, history, disease status, and pre-mobilization laboratory parameters to predict collection success before the start of mobilization. Even without including the peripheral CD34^+^ cell count, that model effectively predicted CD34^+^ cell collection [13].

In our cohort, patient age, the use of a myelotoxic agent, and pre-mobilization platelet count were significantly associated with poor mobilization. A subgroup analysis of patients with hematologic diseases showed the same results, which are consistent with previous studies. Because patients with non-hematologic diseases exhibit different characteristics, especially patient age, with patients with hematologic diseases, a subgroup analysis of patients with non-hematologic diseases was also conducted. These analyses suggest that the risk factors for poor mobilization in patients differ from the risk factors in patients with hematologic diseases. These differences may stem from characteristics associated with non-hematologic diseases but could also be influenced by other factors such as patient demographics (e.g., age and sex) and variations in treatment protocols like chemotherapy regimens and mobilization strategies.

To the best of our knowledge, only a few previous studies considered risk factors for poor mobilization in patients with non-hematologic diseases [20,21]. In those studies, disease status and a history of radiation were identified as risk factors for poor mobilization. Although radiotherapy is safe for cancer patients, caution is warranted in relation to local radiotherapy for symptomatic sites due to the risk of bone marrow aplasia [22]. Furthermore, in cases such as malignant embryonal brain tumors or germ cell tumors, prophylactic craniospinal irradiation can be an indicator for preventing disseminated diseases. However, extended field radiotherapy involving bone marrow has been linked to poor harvest outcomes because of the destruction of sinusoidal vessels and the replacement of hematopoietic stem cells by adipocytes [23]. Consistent with these findings, our study also identified a previous history of radiation therapy as the only factor associated with poor mobilization in patients with non-hematologic diseases [24].

Plerixafor, a CXCR4 antagonist, has been used in combination with G-CSF for effective mobilization. The efficacy of plerixafor as an upfront administrator for PBSCH has been demonstrated, as has its use as a rescue agent when mobilization with G-CSF fails. Although the data vary among study populations, the use of plerixafor is associated with successful mobilization in 68–100% of patients [25,26]. In our patient cohort, 14.1% of patients who received plerixafor suffered PBSCH failure, which is consistent with previous reports. In the poor mobilization group, among patients receiving plerixafor, there were significant differences in hemoglobin level, platelet count, and mobilization regimen. Further study is necessary to specify and confirm the predictive factors for poor mobilization in patients who are administered plerixafor.

This study has some limitations. Relatively few patients were included in the subgroup analyses, and this limited the statistical power. Additionally, the lack of standardization in the mobilization chemotherapy regimen prevented us from comparing the efficacy of mobilization chemotherapy with G-CSF alone. Furthermore, a previous report showed that daratumumab, a monoclonal antibody of CD38 that has been used for patients with MM, is associated with low PBSCH efficiency [27]. However, although patients with MM were included in our cohort, the association with daratumumab could not be assessed due to the small number of patients who received it.

In conclusion, this study identified the potential risk factors of poor mobilization in patients with hematologic diseases and non-hematologic diseases, as well as treatment with plerixafor that was not previously established. For patients eligible for autologous PBSCT, special consideration should be given to mobilization strategy selection to preemptively avoid risk factors for poor mobilization.

## Figures and Tables

**Table 1:** Characteristics of patients who underwent autologous peripheral blood stem cell harvest.

Factors	Poor (*n* = 150)	Good (*n* = 341)	*p*
Age at diagnosis, years	60 (53–64)	57 (32–62)	<0.001
>20	144 (96.0)	273 (80.1)	<0.001
>60	74 (49.3)	107 (31.4)	<0.001
Diagnosis			
Hematologic	144 (96.0)	275 (80.6)	<0.001
Non-hematologic	6 (4.0)	66 (19.4)	
Sex			
Male	76 (50.7)	198 (58.1)	0.139
Female	74 (49.3)	143 (41.9)	
Weight	63.5 (54.0–71.5)	62.1 (54.0–72.1)	0.012
Number of chemotherapy cycles	5 (4–7)	4 (4–6)	0.023
Prior use of myelotoxic agents	102 (68.0)	103 (30.2)	<0.001
Radiotherapy (spine/pelvis)	14 (9.3)	20 (5.9)	0.178
Bone marrow involvement	22 (14.7)	53 (15.5)	0.892
Laboratory parameters *			
Hemoglobin	10.4 (9.1–11.6)	10.6 (9.1–12.2)	0.139
White blood cells	4.05 (2.73–5.09)	4.20 (2.53–5.71)	0.444
Platelets	171 (112–215)	219 (140–288)	<0.001
Diagnosis to mobilization (days)	173 (144–253)	159 (130–200)	0.261
Mobilization regimen			
G-CSF alone	111 (74.0)	227 (66.6)	0.102
Chemotherapy + G-CSF	39 (26.0)	114 (33.4)	
Pre-CD34^+^ cells	5 (2–8)	30 (16–60)	<0.001
Product CD34^+^ cells, first day	0.45 (0.20–0.71)	2.88 (1.79–4.90)	<0.001
Total CD34^+^ cells	3.39 (2.10–4.99)	6.55 (4.66–9.29)	<0.001
Use of plerixafor	134 (89.3)	-	<0.001

* Laboratory parameters were measured on mobilization day 1.

**Table 2:** Logistic regression analysis of factors associated with poor mobilization.

Factor	Univariate		Multivariate	
	OR (95% CI)	*p*	OR (95% CI)	*p*
Age > 20 years	5.978 (2.533–14.110)	<0.001	4.336 (1.131–16.623)	0.032
Sex, female	1.348 (0.917–1.983)	0.129		
Hematologic disease (vs. non-hematologic diseases)	4.919 (2.307–10.491)	<0.001	2.337 (0.603–9.052)	0.219
Weight, >60 kg	1.072 (0.727–1.580)	0.727		
Number of chemotherapy cycles, >6	1.956 (1.234–3.100)	0.004	0.756 (0.394–1.453)	0.401
Myelotoxic agent	4.910 (3.247–7.426)	<0.001	4.093 (2.571–6.516)	<0.001
Radiotherapy (spine/pelvis)	1.652 (0.811–3.367)	0.167		
Bone marrow involvement	0.934 (0.545–1.601)	0.804		
Hemoglobin on mobilization day 1, <10.5 g/dL	1.280 (0.870–1.883)	0.210		
White blood cells on mobilization day 1, <4000/μL	1.141 (0.777–1.676)	0.501		
Platelets on mobilization day 1, <150,000/μL	1.655 (1.105–2.478)	0.015	2.687 (1.624–4.445)	<0.001
Diagnosis to mobilization, >200 days	1.905 (1.262–2.877)	0.002	1.331 (0.776–2.281)	0.299
Mobilization with chemotherapy + G-CSF (vs. G-CSF alone)	0.700 (0.456–1.074)	0.102		

**Table 3:** Logistic regression analysis of factors associated with poor mobilization in patients with hematologic diseases.

Factor	Univariate		Multivariate	
	OR (95% CI)	*p*	OR (95% CI)	*p*
Age > 60 years	1.660 (1.105–2.493)	0.015	1.655 (1.049–2.611)	0.030
Sex, female	1.328 (0.886–1.990)	0.169		
Diagnosis				
Non-Hodgkin lymphoma	2.617 (1.727–3.965)	<0.001	1.947 (0.593–6.391)	0.272
Multiple myeloma	0.437 (0.288–0.663)	<0.001	1.014 (0.303–3.385)	0.983
Weight, >60 kg	1.320 (0.870–2.001)	0.191		
Number of chemotherapy cycles, >6	2.182 (1.3277–3.589)	0.002	0.557 (0.275–1.127)	0.104
Myelotoxic agent	4.749 (3.070–7.348)	<0.001	4.384 (2.681–7.168)	<0.001
Radiotherapy (spine/pelvis)	1.434 (0.641–3.208)	0.381		
Bone marrow involvement	1.171 (0.654–2.097)	0.596		
Hemoglobin on mobilization day 1, <10.5 g/dL	1.827 (1.216–2.747)	0.004	0.988 (0.556–1.628)	0.878
White blood cells on mobilization day 1, <4000/μL	1.702 (1.131–2.562)	0.011	0.988 (0.595–1.640)	0.962
Platelets on mobilization day 1, <150,000/μL	2.987 (1.881–4.744)	<0.001	2.106 (1.205–3.682)	0.009
Diagnosis to mobilization, >200 days	1.957 (1.266–3.025)	0.003	1.520 (0.862–2.682)	0.148
Mobilization with chemotherapy + G-CSF (vs. G-CSF alone)	1.295 (0.795–2.108)	0.299		

**Table 4:** Logistic regression analysis of factors associated with poor mobilization in patients with non-hematologic diseases.

Factors	Univariate	
	OR (95% CI)	*p*
Age > 10 years	5.765 (0.968–34.348)	0.054
Sex, female	0.374 (0.041–3.395)	0.382
Weight, >30 kg	5.333 (0.898–31.680)	0.066
Number of chemotherapy cycles, >6	1.700 (0.283–10.206)	0.562
Myelotoxic agent	0.900 (0.096–8.423)	0.926
Radiotherapy (spine/pelvis)	12.200 (1.934–76.956)	0.008
Bone marrow involvement	0.533 (0.058–4.883)	0.578
Hemoglobin on mobilization day 1, <9 g/dL	0.435 (0.081–2.343)	0.332
White blood cells on mobilization day 1, <3500/μL	0.179 (0.031–1.013)	0.052
Platelets on mobilization day 1, <130,000/μL	2.174 (0.238–19.822)	0.491
Diagnosis to mobilization, >200 days	1.441 (0.242–8.587)	0.688
Mobilization with chemotherapy + G-CSF (vs. G-CSF alone)	0.410 (0.040–4.223)	0.454

**Table 5:** Characteristics of patients who used plerixafor and underwent autologous peripheral blood stem cell harvest.

	Poor (*n* = 19)	Good (*n* = 115)	*p*
Age at diagnosis, years	61 (58–67)	60 (53–64)	0.645
Sex			
Male	12 (63.2)	57 (49.6)	0.327
Female	7 (36.8)	58 (50.4)	
Weight	63.3 (53.5–69.1)	64.8 (55.8–72.6)	0.536
Number of chemotherapy cycles	6 (4–10)	5 (4–6)	0.208
Myelotoxic agent	14 (73.7)	81 (70.4)	1.000
Radiotherapy (spine/pelvis)	0 (0.0)	11 (9.6)	0.363
Bone marrow involvement	1 (5.3)	16 (13.9)	0.465
Laboratory parameters *			
Hemoglobin	9.2 (8.1–10.7)	10.5 (9.4–11.8)	0.015
White blood cells	3.21 (1.58–5.07)	4.15 (2.91–5.09)	0.216
Platelets	126 (59–201)	175 (121–225)	0.016
Diagnosis to mobilization (days)	174 (146–287)	173 (144–229)	0.912
Mobilization regimen			
G-CSF alone	9 (47.4)	95 (82.6)	0.002
Chemotherapy + G-CSF	10 (52.6)	20 (17.4)	
Pre-CD34^+^ cells	1 (0–3)	6 (3–8)	0.004
Product CD34^+^ cells, first day	0.07 (0.05–0.23)	0.50 (0.30–0.74)	<0.001
Total CD34^+^ cells	0.79 (0.27–1.46)	4.14 (3.13–5.65)	<0.001

* Laboratory parameters were measured on mobilization day 1.

## Data Availability

The de-identified data are available from the corresponding authors upon reasonable request.

## Supplementary Materials

The following supporting information can be downloaded at: https://www.mdpi.com/article/10.3390/cancers16101821/s1, Table S1: Mobilization chemotherapy regimen; Table S2: Detailed diagnosis of patients with hematologic diseases; Table S3: Detailed diagnosis of patients with non-hematologic diseases; Table S4: Detailed characteristics of patients with hematologic and non-hematologic diseases.
